# The Role of Dental Arch Dimensions and Impacted Third Molars on Mandibular Anterior Segment Crowding

**DOI:** 10.1055/s-0044-1785186

**Published:** 2024-05-14

**Authors:** Mimoza Selmani, Manushaqe Selmani Bukleta, Shkelzen B. Duci

**Affiliations:** 1 AAB College, Faculty of Dentistry, Prishtina, Kosovo; 2 College of Medical Science, Faculty of Dentistry, “Rezonanca” Pristina, Kosovo; Dental Clinic, Mdent Family Dentistry, Prishtina, Kosovo; 3 Clinic of Plastic Surgery, University Clinical Center of Kosovo, Kosovo

**Keywords:** arch, crowding, dimensions, third molars

## Abstract

**Objectives** This study aimed to investigate the potential impact of arch dimensions and unerupted third molars on mandibular anterior segment crowding.

**Materials and Methods** This study included a total of 240 subjects with an average age of 18 years seeking orthodontic treatment. Panoramic radiographs, alginate impressions, and study models were taken for measurements. This study utilized the Ganss ratio to calculate the ratio of retromolar space to the width of the crown of the third molar, along with other measurements such as intercanine and intermolar widths, arch perimeter, and arch length to assess dental arch dimensions.

**Results** We found that the Ganss ratio and angle B values on both the right and left sides were significantly different between the noncrowding and crowding groups, suggesting a potential role for third molars in mandibular anterior segment crowding. Additionally, there was an increase in angle A on both sides in the crowding group, indicating a more acute angle between the anterior teeth. This study also observed a reduction in the retromolar space on the third molars in the crowding group, further supporting the potential role of third molars in mandibular anterior segment crowding.

**Conclusion** The findings of our study provide substantial evidence to suggest that third molars may contribute to mandibular anterior segment crowding. These findings highlight the importance of carefully evaluating dental arch dimensions and the presence of third molars when assessing and treating mandibular anterior segment crowding.

## Introduction

Anterior dental crowding is one of the most common problems encountered in orthodontic practice; the relapse of anterior crowding following the completion of retention period in orthodontically treated patients has provoked much speculation in the dental literature. 1 Crowding occurs when teeth deviate within the same dental arch. Late incisor crowding is a multifactorial phenomenon, but there is a controversial opinion in orthodontics that third molars contribute to the development of malocclusion or relapse after orthodontic treatment. The arches' size and form also have substantial implications in orthodontic treatment planning affecting the space available for dentition and the stability of normally aligned teeth. Leighton and Hunter 2 established a relationship between dental crowding and the morphological characteristics of the mandible. The anterior component of occlusal force is also essential when it comes to late lower arch crowding.

According to Richardson, 3 pressure from the back of the arch is an important cause of late mandibular incisor crowding during the teenage years. Such pressure because of physiologic mesial drift, the anterior component of the occlusion force on mesially inclined teeth, or the presence of a developing third molar may cause forward movement of the buccal teeth, with a shortening of the arch and increased crowding. Lindauer et al stated that American orthodontists and surgeons believe that mandibular molars can cause dental crowding and should be removed. 4 In another study conducted by Husain and Rengalakshmi, it is concluded that third molars may be one of the reasons for mandibular incisor crowding, if not the only one. 5 However, Tüfekçi et al reported that Swedish and US orthodontists do not believe that impacted mandibular third molars can cause lower anterior crowding even with their anterior force. 6 Similarly, Cotrin et al reported that anterior teeth relapse is unrelated to mandibular third molars. 7 Many researchers have aimed to reveal the influence of potential factors on the stability of the obtained orthodontic results. 8 9 10 And yet, we have not been able to single out only one factor responsible for anterior crowding. However, due to the presence of so much controversy and unfruitful results, we aimed to assess whether arch dimension and impacted third molars have a relationship with anterior dental crowding.

Hence, this study aims to determine the extent to which third molars contribute to lower dental arch crowding by assessing their position and angulation and comparing the measurements between a crowding and a noncrowding group. The specific objectives are presented below:

• To measure the dimensions of the lower arch, including arch length, arch width, and arch perimeter.
• To measure the retromolar space to lower third molar crown width (Ganss ratio) on a panoramic radiograph.
• To investigate the position and angulation of lower third molars by measuring angle A and angle B on panoramic radiographs and comparing all measurements between the crowding and noncrowding groups.

## Materials and Methods

A total of 240 subjects were selected for this study. The sample population was chosen from the patients seeking orthodontic treatment at the Department of Orthodontics. Only this number of patients consented to participate in the study. This cross-sectional study was conducted between October 2018 and October 2019. The sample was divided into two equal groups; 120 participants with Class I normal occlusion and 120 with Class I crowding. All subjects had healthy lower dental arches and angle Class I molar relationship without any artificial crowns or anomalies in crown morphology. Periodontal diseases, decay, and ongoing dental treatments were ruled out.

The authors selected the noncrowding occlusion group from patients who had undergone orthodontic examinations, with all permanent teeth, including erupted third molars, and a normal transversal relationship, leading to optimal intercuspation in both jaws. However, the crowding group had unerupted third molars. The study utilized panoramic radiographs, alginate impressions, and study models. The Ganss ratio was calculated to assess the relationship between dental arch dimensions and mandibular anterior segment crowding. In addition to the Ganss ratio, other measurements were taken, such as intercanine and intermolar widths, arch perimeter, and arch length.

**Fig. 1:**
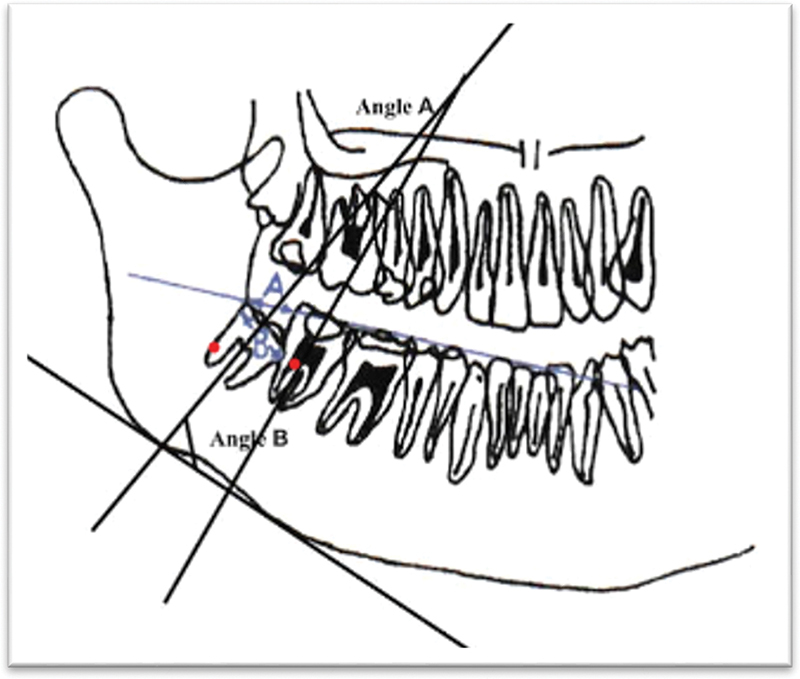
Panoramic radiograph of crowding group (unerupted) lower third molars.

The mandibular dental arch was examined clinically, and each subject had a panoramic radiograph taken using a standardized machine with appropriate criteria and specifications. The overjet and overbite were in normal values (from 2 to 4 mm). The mandibular dental arch was thoroughly examined using a Dentsply Sirona Orthophos E two-dimensional panoramic OPG (orthopantomogram) machine. Alginate impressions were taken to allow the casting of study models, and measurements were made by a single investigator to eliminate interexaminer variability and were assessed at least twice. The reference lines of the panoramic radiographs are presented in Fig. 1 .

### Measurements of Ganss Ratio (the Retromolar Space to the Width of the Crown A/B)

**Fig. 2:**
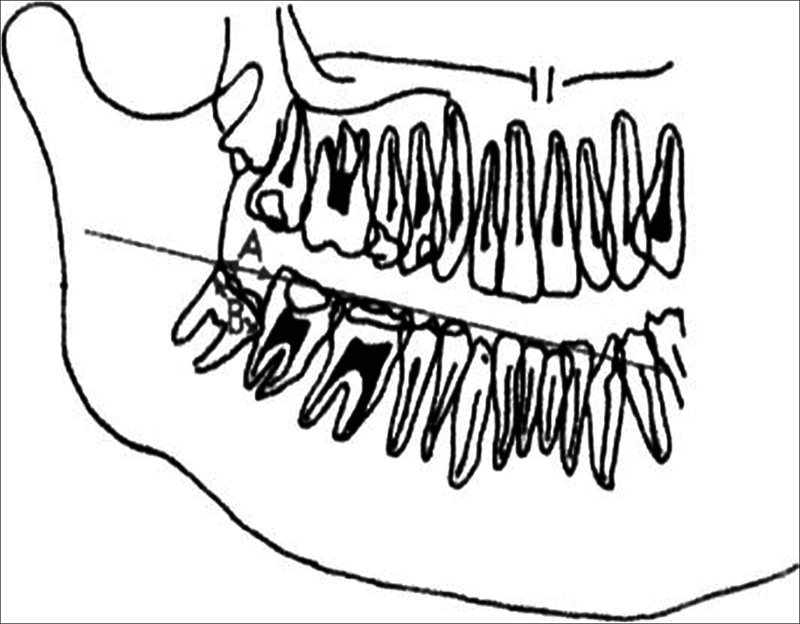
Angle B—third molar angulation to the base of the mandible.

1. The retromolar space to the width of the crown of the third molar (known as the “Ganss ratio”) was measured. First, the width of the third molar crown was measured, and then the distance from the distal surface of the third molar to the anterior border of the ramus of the mandible was measured as a reference point for the retromolar space. The Ganss ratio was then calculated by dividing the retromolar space by the crown width, following the method described by Ganss et al. 11
2. The angle A was measured between the long axis of the mandibular third molar and the long axis of the mandibular second molar. The long axis of each tooth was drawn from the midpoint of the mesial and distal surfaces to the apex of the tooth, which was considered to be the reference point for the measurement. The angle was measured on both the right and left sides of the dental arch. The anatomic landmarks for drawing the lines were the mesial and distal surfaces of the teeth and the apex of each tooth.
3. The angle B measurement was taken by drawing a line from the distobuccal cusp of the lower second molar to the midpoint of the line connecting the mesial and distal tips of the mandibular symphysis. Another line was drawn from the mandibular plane tangent to the lower border of the mandible. The angle between these two lines was measured as the angle B. The anatomic landmarks used for this measurement were the distobuccal cusp of the lower second molar, the midpoint of the line connecting the mesial and distal tips of the mandibular symphysis, and the mandibular plane. The measurement of angle B is shown in Fig. 2 .

Panoramic radiographs, which are routinely taken from orthodontic patients, were selected according to inclusion criteria, second and third molars were traced on overlying acetate paper, and analyzed by a single observer. Mesially inclined third molars were taken into consideration.

### Measurements of the Lower Arch Dimensions

The measurements of the lower arch dimensions on study models were made with the following parameters:

### Arch Length

The measurement of arch dimensions was conducted in segments, which were further categorized into anterior and posterior regions for both the right and left sides. The measurement technique employed was based on the dimensions proposed by Lavelle and Foster, 12 which included three out of the six dimensions. However, the method used by Niedzielska 13 was adopted for measuring the arch width. The measurement of arch length and width was carried out using the Korkhaus caliper.

### Arch Width

To measure the arch width, the Korkhaus caliper was used to take three measurements: intercanine width, interpremolar width, and intermolar width. The intercanine width was measured as the distance between the cusp tips of the canines. The interpremolar width was measured as the distance between the buccal cusps of the first and second premolars. The intermolar width was measured as the distance between the mesiobuccal cusps of the first and second molars.

### Arch Perimeter

The arch perimeter was measured by Lundstrom 14 method on the right and left sides, using a vernier caliper with an accuracy of 0.01 mm. The arch perimeter measurements were made from the distal aspect of the permanent first molar on one side to the distal aspect of the permanent first molar on the other side.

**Fig. 3:**
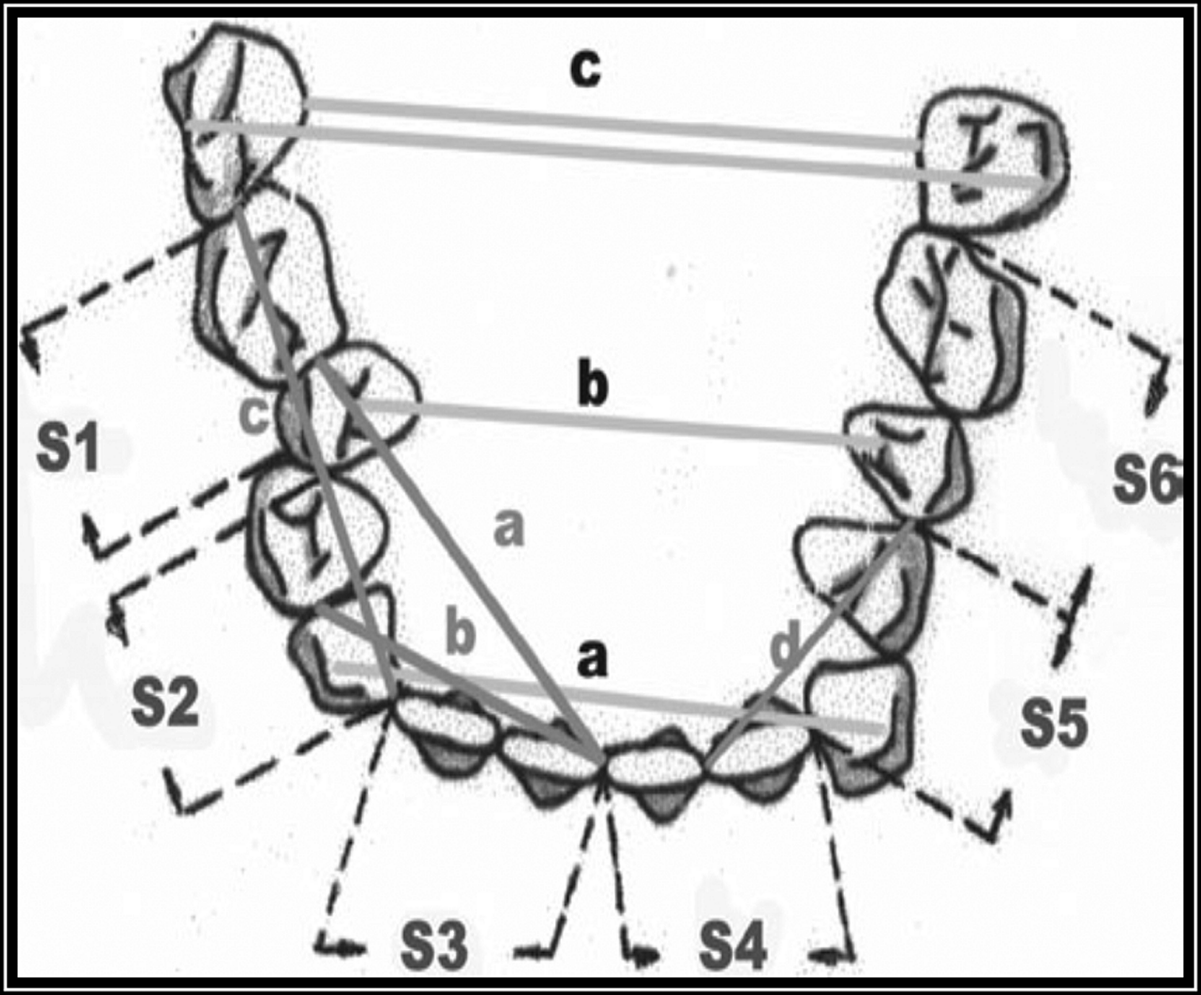
Measurements of lower arch dimensions (arch width a, b, c; arch length a, b, c, d; arch perimeter S1-S6) on study models.

The measurements of lower arch dimensions are presented in Fig. 3 .

### Statistical Analysis

Statistical analysis was conducted with IBM Statistics V.26.00. Statistically significant value was set at *p* -value less than 0.05. Descriptive statistics: Mean, ± 95% confidence interval, were done for Ganss ratio, and the distribution of data in series with numerical characters is tested by the Kolmogorov–Smirnov test, Lilliefors test, and Shapiro–Wilk test. These tests were chosen because of the normal distribution of data. Differences in analyzed parameters between two independent samples (right side and left side)/crowding group and noncrowding occlusion group on the series with numerical characters were tested by the Mann–Whitney U test (Z/U). Correlation between two analyzed parameters was tested by Spearman R coefficient in correlation (R) and correlation between one dependent parameter (S3 & S4) and several independent parameters (Length/a,b,c,d/; Width/a,b,c/; segment/S1, S2, S5, S6/; Ganss Ratio; Angle A; Angle were examined with Multiple Regression Model (R).

## Results

Among the crowding group, 54 (45%) patients were male, and 66 (55%) were female. In contrast, the noncrowding group consisted of 70 (58.33%) males and 50 (41.66%) females. The Pearson chi-squared test was performed, and the *p* -value was more than 0.05 (0.69), indicating no statistically significant difference in the gender distribution between the two groups. The descriptive results of the study are summarized in Tables 1A and 1B .

**Table 1A:** Frequency and percentage distribution of gender

	Crowding group		Noncrowding occlusion group	
Gender	Frequency	Percent	Frequency	Percent
Male	54	45.00	70	58.30
Female	66	55.00	50	41.66
Total	120	100.00	120	100.00
Pearson chi-squared test	0.12			
*p* -Value	0.69			

**Table 1B:** Age distribution among the crowding and noncrowding groups

	Age in years	
	Crowding group	Noncrowding group
Number of subjects	120	120
Mean age	18.05	18.87
Std. deviation	1.57	1.52
Minimum age	16	16
Maximum age	21	21
Pearson chi-squared test	2.75	
*p* -value	0.006	

The mean age of subjects in the crowding group was 18.05 years, with a standard deviation of ± 1.57 years. In contrast, the mean age in the noncrowding group was 18.87 years, with a standard deviation of ± 1.52 years. The difference in mean age between the two groups was found to be statistically significant (Z = − 2.75, *p* = 0.006), with subjects in the noncrowding group being significantly older than those in the crowding group.

Table 2 displays the results of the retromolar space/Ganss ratio and angle B measurements on the right and left sides for participants in the crowding and noncrowding groups. The analysis reveals that the values of the Ganss ratio on the right and left sides of the noncrowding group were significantly higher than those of the crowding group ( *p* < 0.01). Similarly, the values of angle B on the right and left sides of the noncrowding group were significantly greater than those of the crowding group.

**Table 2:** Differences of the retromolar space/Ganss ratio, angle B on the right and left side between crowding and noncrowding groups

Ganss ratio	Rank sum crowding	Rank sum noncrowding	U	Z	*p* -Value
Ganss ratio/R	7284.50	21635.0	24.50	−13.34	0.00
Ganss ratio/L	7300.00	21620.0	40s.00	−13.31	0.00
Angle B/R	10920.0	18000.0	3660.00	−6.58	0.000
Angle B/L	9649.00	19271.0	2389.00	−8.95	0.000

**Fig. 4:**
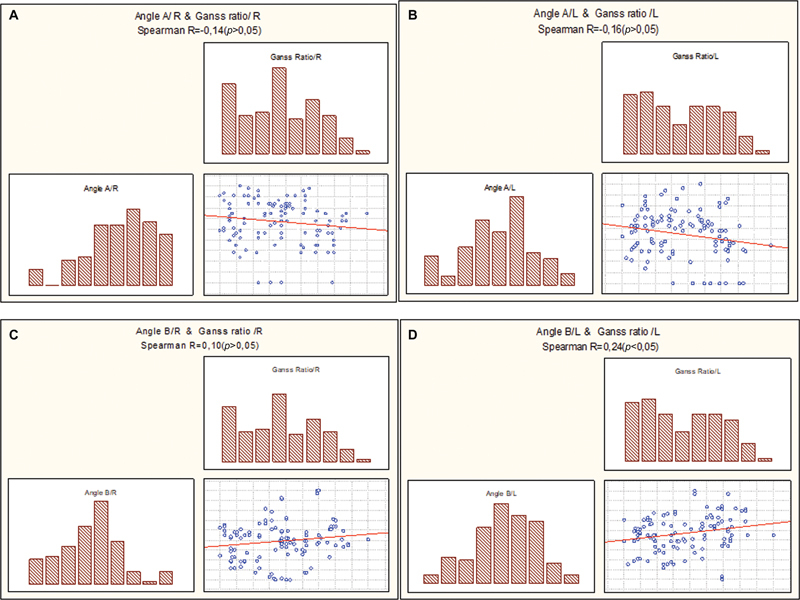
( **A** ) Examined relation with Spearman R coefficient of angle A/R and Ganss ratio/R, ( **B** ) of angle A/L and Ganss ratio/L, ( **C** ) of angle B/R and Ganss ratio/R, and ( **D** ) of angle B/L and Ganss ratio/L.

The Spearman R coefficient analysis revealed a weak and insignificant negative correlation between the right and left sides of the angle A and Ganss ratio. However, the study found that an increase in angle A on either side is associated with a reduction in retromolar space of third molars, as shown in Fig. 4A and B .

On the other hand, a weak negative insignificant correlation was observed between angle B and Ganss ratio/R using the Spearman R coefficient. Nevertheless, a weak positive significant correlation was found between angle B and Ganss ratio/R on the left side, as depicted in Fig. 4C and D . Notably, an increase in angle B on both sides corresponded to an increase in the retromolar space of third molars.

### Multiple Regression Analysis

The multiple regression results are shown in Table 3 . The result reveals that the increase in distance of length b/R, the distance of segment S2 by 1 mm, will significantly increase the distance of the segment (S3). The individual parameter changes in length a/R, length d/R, width a, width c, S1, Ganss ratio/R lead to increase in segment S3. However, the increase is not statistically significant, with a *p* -value greater than 0.05.

**Table 3:** The relationship between the segment of the arch perimeter (S3) as a dependent variable and Ganss ratio/R, angle A/R, angle B/R, length, width, and segments of the lower arch perimeter as an independent variable

	Beta	SE. of beta	Beta	SE of beta	*t* (27)	*p* -Value
Intercept			5.96	1,17	5.11	0.00
Length a/R	0.10	0.11	0.03	0.03	0.96	0.34
Length b/R	0.36	0.10	0.11	0.03	3.71	0.000
Length c/R	−0.07	0.11	−0.02	0.02	−0.68	0.50
Length d/R	0.06	0.12	0.02	0.05	0.54	0.59
Width a	0.001	0.12	0.0003	0.04	0.01	0.99
Width b	−0.05	0.12	−0.009	0.02	−0.37	0.71
Width c	0.15	0.11	0.03	0.02	1.31	0.19
S1	0.09	0.10	0.04	0.05	0.94	0.35
S2	0.22	0.09	0.08	0.03	2.43	0.02
Ganss ratio/R	0.003	0.09	0.01	0.28	0.04	0.97
Angle A/R	−0.03	0.09	−0.002	0.01	−0.34	0.73
Angle B/R	−0.10	0.09	−0.004	0.004	−1.11	0.27

Abbreviation: SE, standard error.

R = 0.59; F(12.107) = 4,73; *p* < 0.000.

Furthermore, the multiple regression results of the relationship between the segment on the left side of the arch perimeter (S4) (dependent variable) and Ganss ratio/L; angle A/L; angle B/L; length; width, segments of the lower arch perimeter (independent variable) are shown in Table 4 .

**Table 4:** The relationship between the segment on the left side of the arch perimeter (S4) as the dependent variable and Ganss ratio/L; angle A/L; angle B/L; length; width, and segments of the lower arch perimeter as an independent variable

Parameter	Beta	SE of beta	B	SE. of beta	*t* (27)	*p* -Value
Intercept			5.03	1.23	4.08	0.00
Length a/L	0.25	0.13	0.09	0.05	1.91	0.06
Length b/L	0.14	0.09	0.06	0.04	1.56	0.12
Length c/L	−0.18	0.12	−0.04	0.03	−1.47	0.14
Length d/L	0.08	0.15	0.03	0.06	0.56	0.58
Width a	−0.01	0.11	−0.004	0.04	−0.10	0.92
Width b	0.21	0.12	0.04	0.02	1.77	0.08
Width c	−0.11	0.12	−0.02	0.02	−0.93	0.36
S5	0.04	0.10	0.02	0.05	0.45	0.66
S6	0.28	0.11	0.16	0.06	2.56	0.01
Ganss ratio/L	0.02	0.09	0.05	0.28	0.19	0.85
Angle A/L	−0.03	0.10	−0.001	0.01	−0.27	0.79
Angle BL	−0.02	0.10	−0.001	0.004	−0.24	0.81

Abbreviation: SE, standard error.

From the result, the individual changes of parameters—angle A/L; angle B/L; length c/L; width a; width c—lead to a reduction in segment S4; however, the reduction is not statistically significant.

Furthermore, the increase in distance in segment S6 for 1 mm leads to an increase in the distance of segment S4 by 0.16 mm (B = 0, 16), thus making it statistically significant with a *p* -value of 0.01.

## Discussion

Dental crowding is a common problem in orthodontic clinics and is defined as a discrepancy between the space available and the space required in dental arches. 15 The etiology of crowding is multifactorial, and it has been suggested that tooth size, arch dimension, unerupted molars, and environmental factors play a role in its development. 16 17 Many researchers have investigated the relationship between crowding and these parameters in detail. 18 19

While crowding can affect the entire arch, it is more commonly localized to the anterior segment of the lower arch. 20 The cause of this anterior crowding is still under debate; however, it has been suggested that it may be due to the inadequate growth of the mandible or an imbalance between tooth size and arch dimension. 21 Whether impacted molars affect dental crowding is still highly controversial. 22 A study conducted in China has revealed that mesiodistally angulated molars impact anterior teeth crowding. 23

According to Howe et al, 24 the authors found that arches with crowding were shorter than those without crowding. This was confirmed in our study, and it was observed that the crowded group's arch was shorter than the noncrowded group's. This may be attributed to the fact that crowded teeth rotate and tip, leading to decreased available arch space. This can cause the arch to become narrower, shorter, and more constricted, which can contribute to crowding. However, it is essential to note that the difference in arch length between crowded and noncrowded arches in the study was not significant for all parameters, indicating that other factors may also be involved in the development of crowding. As a result, we cannot establish a direct causative relationship between arch length and dental crowding.

In the works of Waheed-ul-Hamid and Imran, 25 arch length was found to be greater in noncrowded arches as compared to the crowded group. This is in correlation with the findings of this study because, in the noncrowding group, the length of the lower arch is significantly greater in relation to the crowding group. These findings support the importance of considering arch length as a contributing factor to dental crowding. In addition, a study by Muhammed et al 21 showed that tooth size discrepancies are a significant factor in dental crowding. This highlights the complex interplay between various dental parameters in the etiology of dental crowding.

Research has shown that dental crowding is more commonly observed in the anterior segment of the dental arch due to several factors. The anterior teeth are more susceptible to tipping and rotation, leading to misalignment and crowding in the anterior segment. 26 27 The individual changes in the retromolar region and arch dimensions were crucial in reducing or increasing the anterior segment of the lower arch. 17 This suggests that variations in arch dimensions may contribute to the development of dental crowding. Moreover, other studies have highlighted the importance of considering factors such as tooth size, arch width, and depth in evaluating dental crowding. 28 29

Lateral cephalometric radiographs have been used by several researchers to assess the position of third molars, but they are not the most accurate radiographs due to the significant amount of superimposition present. 26 29 OPGs are considered more reliable radiographs for assessing the position and angulation of third molars. Therefore, the authors of this study opted to use panoramic radiographs to evaluate the angulation and position of third molars and to measure the space/third molar width ratio.

Hattab and Alihaija 28 found that the space/third molar width ratio was significantly more prominent in the group of erupted third molars than in the impacted group. This is consistent with the findings of the current study, where the noncrowding group (erupted third molars) had significantly larger values of the space/third molar width ratio than the crowded group (unerupted third molars). Zachrisson 30 noted that mandibular incisor crowding is often caused by a developing mandibular third molar with insufficient space, and a mesially directed force can contribute to early mandibular arch crowding. This is consistent with our study, as the authors found an insufficient retromolar space in the crowding group, where the Ganss ratio values were smaller than in the noncrowding group.

Niedzielska et al 31 suggested that the ratio of the third molar angle to the base of the mandible and the third molar angle to the second molar inclination can be used to predict the position of the lower third molar in the dental arch, facilitating a decision to retain or remove it. Therefore, early diagnosis and management of developing third molars are important to prevent crowding and reduce the need for extraction.

This study provides compelling evidence of a significant association between lower arch crowding and third molars. The results demonstrate that the angle between the third molars and the mandibular plane is smaller in the crowding group compared to the noncrowding group. However, we have established that participants who do not have enough space for the proper alignment of their front teeth do not have enough space for wisdom teeth either. Furthermore, the angle between the third and second molars is higher in the crowding group than in the noncrowding group. This suggests that the mesial inclination of third molars may exert mesial force on lower arch teeth.

This study has certain limitations, such as a small sample size and a cross-sectional study design. Due to our study design, we could not establish a direct causative relationship between third molars and mandibular incisor crowding. We suggest extensive cohort and longitudinal study designs, which also exclude other contributing factors.

## Conclusion

According to our study, third molars appear to be a contributing factor to mandibular anterior segment crowding. There are several potential points to consider regarding the difficulties in orthodontic treatment planning and making a timely decision about third molar removal.
